# Contrast-enhanced ultrasound of pediatric lungs

**DOI:** 10.1007/s00247-020-04914-8

**Published:** 2021-05-12

**Authors:** Vasileios Rafailidis, Savvas Andronikou, Hans-Joachim Mentzel, Maciej Piskunowicz, Judy H. Squires, Carol E. Barnewolt

**Affiliations:** 1 Department of Radiology, King’s College Hospital, Denmark Hill, London, SE5 9RS UK; 2 Department of Radiology, Children’s Hospital of Philadelphia, Perelman School of Medicine, University of Pennsylvania, Philadelphia, PA USA; 3 Section of Pediatric Radiology, Institute of Diagnostic and Interventional Radiology, University Hospital, Jena, Germany; 4 Department of Radiology, Medical University of Gdansk, Gdansk, Poland; 5 Department of Radiology, University of Pittsburgh Medical Center, Pittsburgh, PA USA; 6 Department of Radiology, Boston Children’s Hospital, Harvard University, Boston, MA USA

**Keywords:** Abscess, Children, Contrast-enhanced ultrasound, Empyema, Lung, Necrotizing pneumonia, Parapneumonic effusion, Pneumonia, Ultrasound contrast agents

## Abstract

In addition to radiography, ultrasound (US) has long proved to be a valuable imaging modality to evaluate the pediatric lung and pleural cavity. Its many inherent advantages, including real-time performance, high spatial resolution, lack of ionizing radiation and lack of need for sedation make it preferable over other imaging modalities such as CT. Since the introduction of ultrasound contrast agents (UCAs), contrast-enhanced ultrasound (CEUS) has become a valuable complementary US technique, with many well-established uses in adults and evolving uses in children. Lung CEUS applications are still not licensed and are performed off-label, although the added value of CEUS in certain clinical scenarios is increasingly reported. The limited evidence of CEUS in the evaluation of pediatric lungs focuses primarily on community-acquired pneumonia and its complications. In this clinical setting, CEUS is used to confidently and accurately diagnose necrotizing pneumonia and to delineate pleural effusions and empyema. In addition to intravenous use, UCAs can be administered directly into the pleural cavity through chest catheters to improve visualization of loculations within a complex pleural effusion, which might necessitate fibrinolytic therapy. The purpose of this paper is to present the current experience on pediatric lung CEUS and to suggest potential additional uses that can be derived from adult studies.

**Supplementary Information:**

The online version contains supplementary material available at 10.1007/s00247-020-04914-8.

## Introduction

Pediatric community-acquired pneumonia represents a major cause of lung pathology in children, occurring with an approximate annual incidence of 14.4–15.7 per 10,000 in children younger than 16 years [1, 2]. Its incidence is highest among younger children, with a frequency of up to 33.8 per 10,000 in those younger than 5 years and up to 62.2 per 10,000 in those younger than 2 years [1, 2]. Community-acquired pneumonia can be complicated by parapneumonic effusion, empyema, lung parenchymal necrosis and abscess formation [3].

Several terms are used to describe pleural complications of pneumonia. A pleural effusion is fluid in the pleural space when no pneumonia is present, while a parapneumonic effusion is always associated with an adjacent consolidation. The term simple parapneumonic effusion refers to an exudative collection without internal septations, while a complex parapneumonic effusion contains fibrinous strands and septations. Empyema is used to describe a pus-containing pleural collection [4]. Necrotizing pneumonia is a rare but severe complication of community-acquired pneumonia characterized by necrosis and liquefaction of consolidated lung tissue, and this can be complicated by solitary or multiple thin-wall intrapulmonary cavities [5]. Lung abscess refers to suppuration of lung tissue and formation of a cavity that contains necrotic debris or gas and is usually surrounded by a well-formed thick-wall inflammatory capsule [6]. Lung necrosis and lung abscess represent a continuum in the spectrum of microbial lung infections. Parapneumonic effusions and empyema can affect approximately 12.6 per 10,000 children that are admitted with pneumonia but are more common in infants and young children [7]. The rate of necrotizing pneumonia among all patients with community-acquired pneumonia who require hospital admission is estimated at 0.8–7.0% [5, 8].

Imaging plays an important role in the diagnosis and management algorithm of pneumonia. Radiography is the first-line imaging modality to evaluate pediatric lungs and is used to confirm a clinical diagnosis of infection and determine whether antibiotics or additional evaluation is needed [9]. In cases of suspected complications, US is very useful to identify parapneumonic effusions and to guide drainage [4]. CT is usually reserved for further evaluation of complicated lung infections if surgical intervention is planned or if preceding imaging examinations are inconclusive [10].

Contrast-enhanced ultrasound (CEUS) has emerged as a valuable complementary US technique that can offer additional information to gray-scale and Doppler US [11]. There is limited but growing experience with the use of CEUS for the evaluation of complicated pneumonia [12, 13]. Only one small paediatric case series has been published. This study used intravenous (IV) and intracavitary CEUS to confirm the diagnosis of necrotizing pneumonia, to improve delineation of a parapneumonic effusion, and to guide decision-making in cases of multi-loculations [12].

For other lung pathologies, the role of CEUS has been explored in adults: to confirm or exclude pleuropneumonia in patients with clinical signs of pleurisy and indeterminate focal pleural lesions, to differentiate between compressive and obstructive causes of atelectasis, to characterize sub-pleural pulmonary lesions and to assist interventional procedures [14–19]. There is also some limited experience using CEUS to evaluate peripheral pulmonary lesions in patients with pulmonary embolism [20]. These adult studies described the CEUS method to evaluate the lungs and provide important information about the timing and enhancement pattern of various lung lesions, which can aid the interpretation of imaging findings.

The aim of this review article was to present the current experience with CEUS in pediatric lung applications, with emphasis on the examination technique and interpretation of the imaging findings in pediatric pneumonia. We also discuss the established experience from adult studies to highlight procedural recommendations and to suggest potential future uses in children.

## Contrast-enhanced ultrasound examination technique

### Pre-contrast scan

Pediatric lung CEUS examination starts with gray-scale US complemented with color/power Doppler assessment of the pleural cavity and adjacent lung parenchyma. Because the air contained within the lung does not allow transmission of US, only pleural-based or peripheral lung parenchymal lesions and areas of consolidation can be assessed. Either a convex transducer of 3–8 MHz or a high-frequency linear transducer of 7–12 MHz can be used, depending on the size and depth of the area of interest. The child can be examined in a seated, supine or lateral decubitus position. Scanning should be performed from anterior, lateral and posterior intercostal spaces. In addition to intercostal spaces, subcostal, suprasternal and parasternal acoustic windows are useful for thoracic US and subsequent CEUS performance [21–23].

### Ultrasound contrast agent dose

SonoVue (Bracco Imaging, Milan, Italy), which is marketed as Lumason (Bracco Diagnostics, Monroe Township, NJ) in the United States, is the only ultrasound contrast agent (UCA) that has been used for lung CEUS applications in adults and children [12–20, 24–30]. In adults, SonoVue doses range from 2.4 mL to 4.8 mL for various lung-related IV CEUS applications [14–17, 26, 30]. In 2016, the United States Food and Drug Administration (FDA) approved Lumason for IV use in pediatric liver imaging. The approved dose has been adapted to the child’s body weight, with a recommended dose of 0.03 mL per kg with a maximum limit of 2.4 mL per injection. When it comes specifically to pediatric lung CEUS, SonoVue/Lumason is currently used off-label. Prior to FDA approval of Lumason, one pediatric study on lung CEUS reported a dosage scheme for SonoVue that was based on the child’s age: 0.6 mL for children <6 years old, 1.2 mL for children 6–12 years and 2.4 mL for children >12 years of age [12].

Lung CEUS imaging can also be done following the administration of the UCA directly into the pleural space through drain catheters [12]. This is useful to confirm that the drain is in the correct position and to assess for communicating loculations or septations. For this application, a small amount of UCA is diluted into normal saline and subsequently administered via the chest drain. One pediatric study suggested that 0.1 mL of the UCA can be diluted into 20 mL of normal saline for this purpose [12].

### Intravenous contrast-enhanced ultrasound examination technique

After the UCA is injected, microbubbles are quickly evident within the lung; enhancement starts even sooner in neonates because of their faster heart rates compared to older children. The primary goal of IV CEUS is to describe the enhancement characteristics of the examined region. Normal lung parenchyma shows homogeneous enhancement. Atelectatic lung also enhances as the lung parenchyma is vascularized. However, avascular regions that result from necrotizing pneumonia or abscess formation remain non-enhancing [12, 15].

In addition, CEUS can provide information about the timing of enhancement as an indicator of the origin of vascularization. Lung has a dual arterial supply: the pulmonary and bronchial arteries [31]. The pulmonary circulation transports deoxygenated blood from the right heart to a branching network of pulmonary capillaries that extend to the level of the alveolar ducts. The bronchial circulation arises from the aorta and intercostal arteries, providing oxygenated blood to the bronchi and visceral pleura.

Because the pulmonary arterial flow comes from the right heart and the bronchial arteries are fed by the left heart by way of the aorta, the timing of microbubble appearance in a lung lesion can indicate which type of artery is supplying the in-flow. In healthy adults, microbubbles normally appear in the right heart 1–5 s after IV injection, while it takes 8–11 s for them to appear in the left heart. Therefore, contrast enhancement that appears within the lung from 2 s to 6 s suggests pulmonary arterial origin, whereas contrast appearing later than 6 s is likely from blood flow through systemic bronchial arteries [15–17]. Exceptions to this rule include cardiac failure, chronic pulmonary disease, or metabolic conditions, all of which can prolong the arrival time of microbubbles in the pulmonary and bronchial arterial circulation. The combined pulmonary and bronchial arterial phases last up to 30 s, followed by the parenchymal phase, which takes place up to 5 min after contrast injection [15–17].

The onset of enhancement of a lung lesion can also be compared visually to adjacent organs and body regions, such as the spleen, liver and the chest wall. As a general principle, if contrast agent is visible in a lung lesion before it appears in the adjacent organs/body regions, then the lesion is characterized as having early enhancement from pulmonary arterial supply, whereas lung lesions that demonstrate contrast agent at the same time as the reference organs/body regions are considered to have systemic bronchial artery blood supply [17].

Similar to the onset of enhancement, the duration of contrast enhancement within a lung lesion can be evaluated based on the time that contrast agent disappears (washes out) from the lesion as compared to adjacent organs (e.g., liver and spleen). If contrast agent washes out from the lung lesion prior to the adjacent organs (<120 s), then the lesion is characterized as having early washout, whereas if there is sustained enhancement within the lesion after the contrast agent has washed out from the adjacent organs (>120 s), the lesion is characterized as having prolonged enhancement [18].

## Pneumonia in children

### The role of gray-scale and Doppler ultrasound in pediatric pneumonia

When it comes to pediatric lung imaging, the relatively unossified costochondral and sternal cartilage of the neonate and infant, and the small amount of superficial adipose tissue in pediatric patients allows deeper tissue penetration and favorable acoustic windows. Because of these features, US has been established as an optimal modality for imaging diaphragmatic, pleural and chest wall lesions [21–23]. In addition, in children there is a reduced distance between the transducer and the lung parenchyma or pleural space, allowing for high-spatial-resolution imaging.

US is already well-established for the characterization of opacities that have been detected by chest radiograph. US can accurately differentiate whether the opacity is from atelectasis or subpleural consolidation, pleural disease, or a variable combination of these entities [21, 22, 24, 32–34].

When air in the lung is replaced by fluid and pus, the sonographic appearance of the lung parenchyma is similar to the echotexture of the adjacent liver; thus the term “lung hepatization” is frequently used to describe this echotexture pattern. Because the air is displaced, US can effectively evaluate consolidation that reaches the pleural surface or diaphragm [21–23, 35]. The remaining bronchial air manifests as the sonographic air-bronchogram, which appears as hyperechoic, dot-like, and branching linear structures that might be mobile [36]. If the bronchi are filled with fluid or mucus, a hypoechoic bronchogram might become visible. Color Doppler can readily differentiate this bronchogram from branching blood vessels [21, 37]. Observing the movement of the air within bronchi is a useful sign to differentiate atelectasis from pneumonia. A moving air bronchogram usually indicates pneumonia, whereas a static air bronchogram is most often seen in atelectasis [22, 37].

In case of pneumonia-related complications, US can accurately depict a parapneumonic effusion and quantify its volume, easily detecting effusions as small as 3–5 mL [38]. Although it might not be possible to definitively characterize an effusion as transudative or exudative, US is useful for distinguishing between simple and complex fluid. Broadly speaking, US shows simple effusions as anechoic or hypoechoic, unilocular fluid; these are freely mobile during respiration or changes in body position. Exudative collections or hemothorax might contain echogenic debris, and complex collections can appear multi-loculated with internal septations from fibrinous strands [21–23]. Distinguishing between simple and complex fluid can help determine whether a chest tube will effectively drain the effusion or fibrinolytic therapy is indicated. In addition to diagnosis, US is suitable for monitoring collections and consolidation during treatment [4, 21].

Lung necrosis is a dreaded complication of pneumonia. Its presence is accompanied by increased rates of long-term sequelae such as fibrotic scars, pneumatocele and bronchopleural fistula formation [39, 40]. On US, lung necrosis within a region of consolidation appears as ill-defined areas of decreased echogenicity and vascularity. However, heart pulsation and respiratory motion might inhibit Doppler US assessment because motion artifact makes it challenging to confirm presence or absence of vascularity. In addition, using US with Doppler techniques, it might be difficult to differentiate large and complex adjacent effusions with debris and multiple internal septations from lung consolidation and lung necrosis.

### The role of intravenous contrast-enhanced ultrasound in pediatric pneumonia

**Fig. 1:**
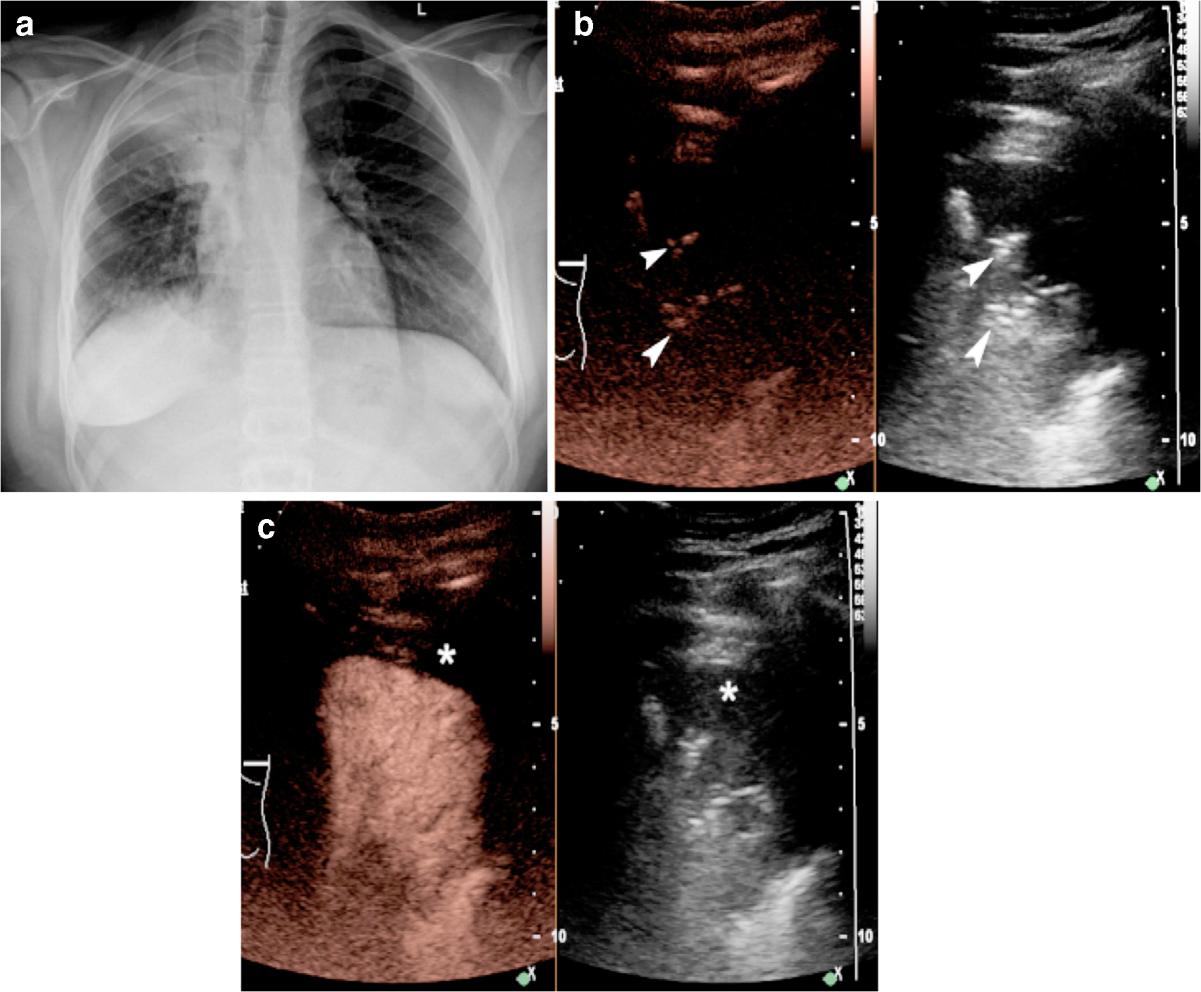
Endocarditis and right-side pneumonia (*Staphylococcus aureus*) in a 14-year-old girl. **a** Posteroanterior chest radiograph demonstrates right upper lobe consolidation containing branching air-bronchogram. **b** Baseline pre-contrast US scan in dual-screen mode with simultaneous display of contrast mode (*left*) and gray-scale mode (*right*) before contrast agent injection. Coronal plane. Within the area of lung consolidation, highly echogenic branching structures (*arrowheads*) appear similar in contrast and reference gray-scale images, suggestive of air inside the bronchi (ultrasonographic air-bronchogram sign). **c** Contrast-enhanced ultrasound (CEUS) following intravenous administration of contrast agent. Dual-screen mode with simultaneous display of contrast mode (*left*) and gray-scale mode (*right*) in coronal plane. The consolidated lung demonstrates uniform enhancement, excluding the diagnosis of necrotizing pneumonia. Note a small anechoic parapneumonic effusion (*asterisk*)

**Fig. 2:**
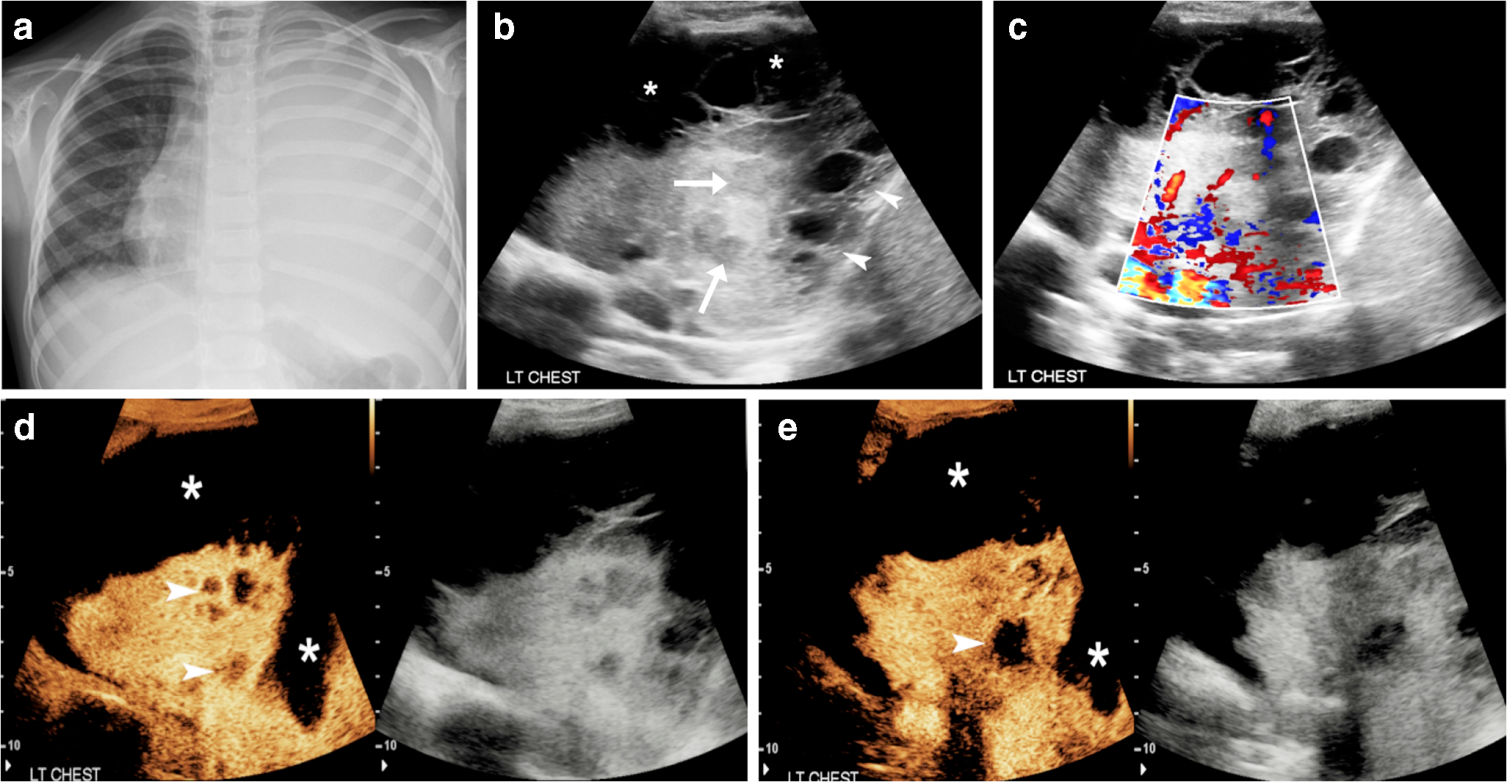
Complicated pneumonia in a 5-year-old girl. **a** Posteroranterior chest radiograph shows complete opacification of the left hemithorax, with no visualization of the left hemidiaphragm and the left cardiac border. The cardiac silhouette has been displaced to the right. **b** Gray-scale US in coronal plane through the left lung shows consolidated lung parenchyma with heterogeneous appearance containing some ill-defined hypoechoic areas (*arrows*). Adjacent to the consolidated lung there is complex multiloculated pleural effusion (*asterisks*), in keeping with empyema, which extends in a sub-pulmonary location (*arrowheads*). It is difficult to delineate the exact extent of the empyema and evaluate the adjacent consolidated lung because of the complex echogenicity of the empyema. **c** Color Doppler US in coronal plane through the left lung. Image quality is degraded by overwriting artifacts from movement and breathing. There is some blood flow signal within the consolidated lung; however, exclusion of necrotizing pneumonia is not possible. **d, e** Contrast-enhanced ultrasound (CEUS) dual-screen mode with simultaneous display of contrast (*left*) and gray-scale (*right*) modes from superior (**d**) to inferior (**e**) aspects of the left hemithorax in coronal plane. There is heterogeneous enhancement of the consolidated lung, which is clearly distinguished from the adjacent non-enhancing parapneumonic effusion. Within the consolidated lung there are multifocal non-enhancing areas (*arrowheads*) suggestive of avascular lung tissue that is not surrounded by thickened or rim-enhancing wall, in keeping with necrotizing pneumonia rather than abscesses. CEUS also significantly improved the delineation of the overall volume of the parapneumonic effusion (*asterisks*)

In the setting of pediatric complicated pneumonia, CEUS can play an important role in the diagnostic and management algorithms. Similar to other organ applications, CEUS provides real-time observation of tissue enhancement pattern. Furthermore, it is an ideal modality for children because it obviates the need for sedation or ionizing radiation. Besides its safety advantages, CEUS can improve the diagnostic performance of gray-scale and Doppler US techniques to accurately detect areas of necrotizing pneumonia within lung consolidations, delineate their exact extent, and clearly differentiate them from adjacent complex pleural effusions (Figs. 1 and 2).

**Fig. 3:**
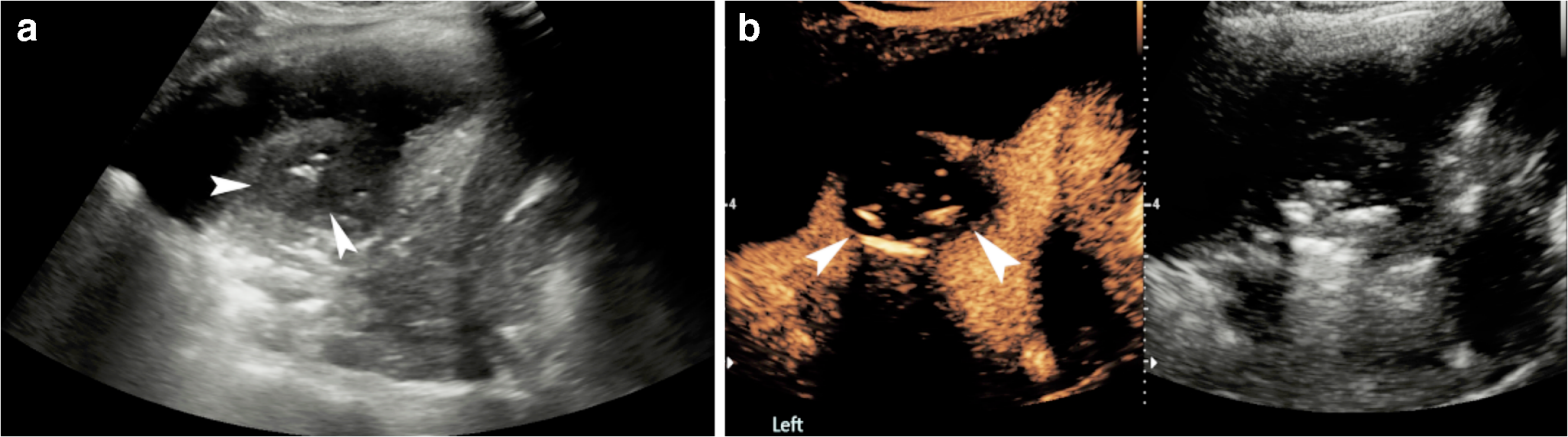
Worsening symptoms in a 2-year old girl hospitalized with community-acquired pneumonia. **a** Gray-scale US of the left lung in coronal plane shows a heterogeneous appearance of the visualized lung parenchyma in the left lung base, with a round, relatively hypoechoic region (*arrowheads*) centrally within the consolidated lung, containing some echogenic foci, attributable to air bronchogram. Adjacent to the consolidation there is a large volume of pleural effusion, with no significant loculations or debris. **b** Contrast-enhanced ultrasound (CEUS) dual-screen mode with simultaneous display of contrast mode (*left*) and gray-scale mode (*right*), following intravenous administration of US contrast agent. Coronal view of the left lung shows enhancement of the consolidated lung, but the central area shows absence of enhancement (*arrowheads*) with no definite wall or rim enhancement, suggestive of necrotizing pneumonia

**Fig. 4:**
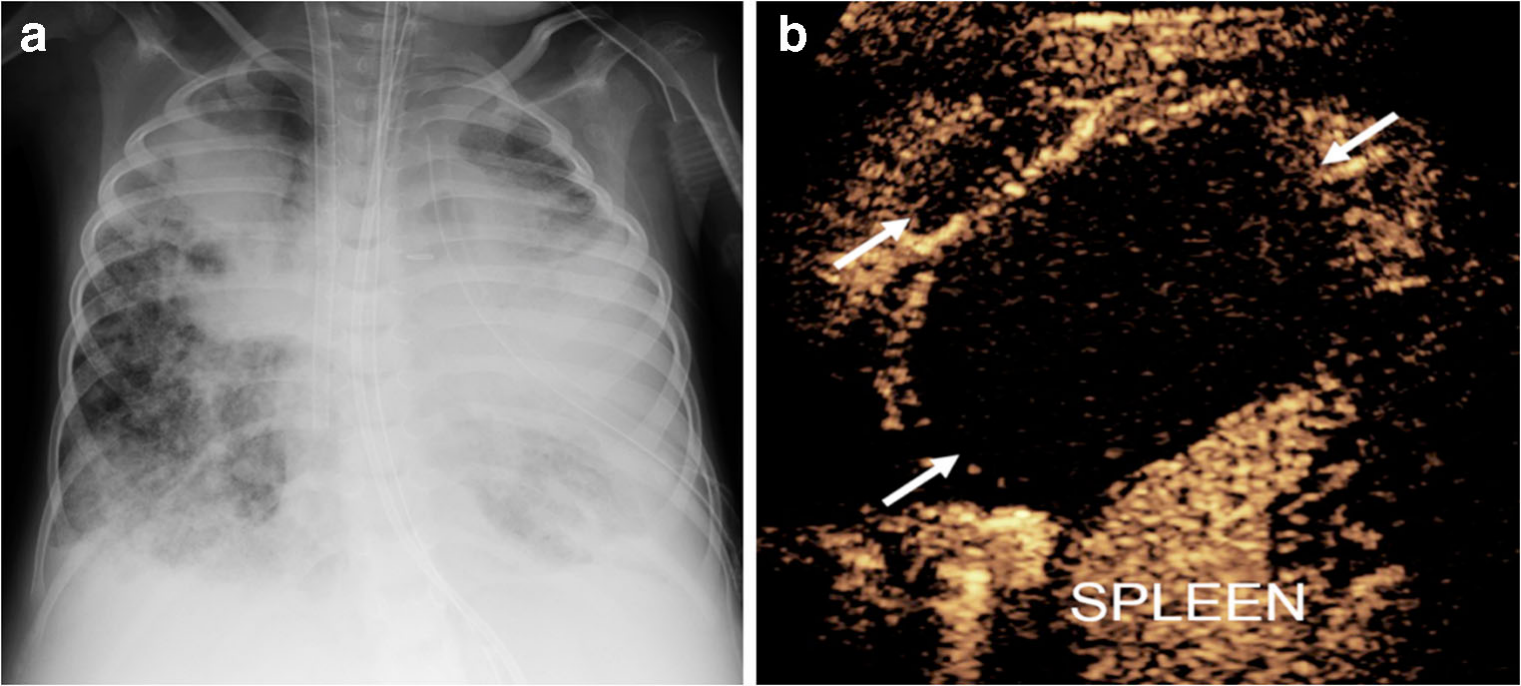
Lung infection and concern for lung abscess in a 6-year-old boy with chronic renal and respiratory failure caused by prune belly syndrome, on extracorporeal life support. **a** Posteroanterior chest radiograph demonstrates extensive multifocal opacities throughout both lungs, more confluent in the right upper lobe and left mid and lower lung lobes. **b** Contrast-enhanced ultrasound (CEUS), coronal view of the left lung. Following intravenous administration of contrast agent, there is a large non-enhancing area (*arrows*) within the left lower lobe with a smaller similar non-enhancing area anteriorly. These findings are in keeping with necrotizing pneumonia

**Fig. 5:**
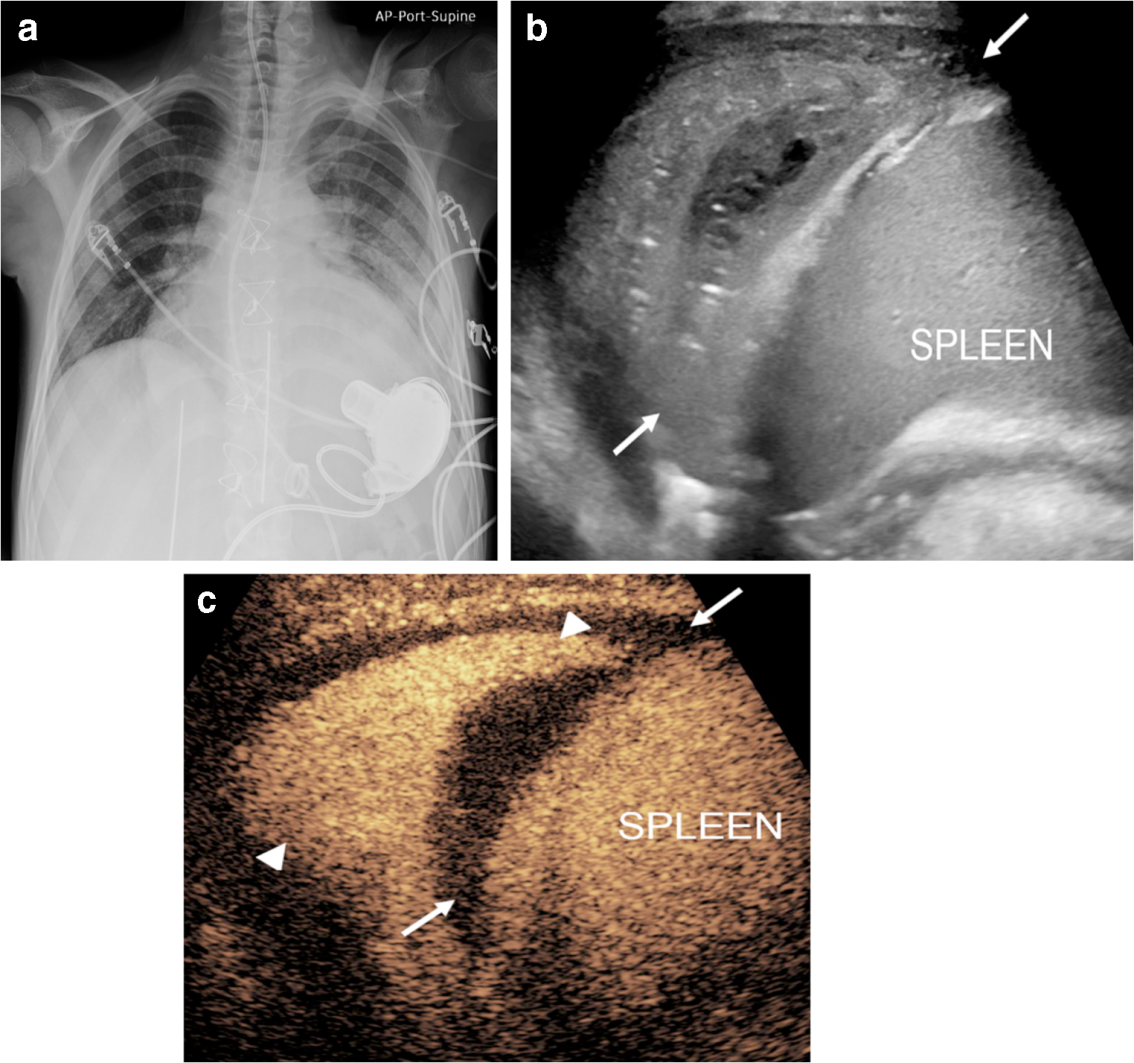
Lung infection and clinical concern for necrotizing pneumonia versus complex pleural fluid in 15-year-old boy with heart failure requiring left ventricular assist device, presenting pre heart transplant. **a** Posteroanterior chest radiograph demonstrates cardiomegaly with only minor left basilar ill-defined opacities. **b** Gray-scale US, coronal view of the left lung base, shows abnormal appearance of the left lung base (*arrows*) with consolidated lung parenchyma containing some scattered air foci. Ill-defined hypoechoic area is noted in the lung base. It is difficult to differentiate whether this represents an abscess within the lung parenchyma or an adjacent complex pleural effusion. **c** Contrast-enhanced ultrasound (CEUS) in contrast-only mode, coronal plane, shows homogeneously enhancing lung parenchyma (*arrowheads*) in the left lung base and non-enhancing pleural fluid (*arrows*) surrounding the left lung. CEUS findings excluded the diagnosis of necrotizing pneumonia and confirmed the presence of pleural empyema

A study conducted in 10 children with complicated pneumonia showed that CEUS can outperform US in the diagnosis of necrotizing pneumonia and improve the operator’s diagnostic confidence by increasing conspicuity of the pathology [12]. In this study, all children (ages 1–12 years) undergoing CEUS examination presented with complicated pneumonia, including either necrotizing pneumonia or a parapneumonic effusion in the form of a simple pleural effusion or empyema. It was found that IV CEUS accomplished a more comprehensive evaluation of both the consolidated lung parenchyma and adjacent pleural effusion. CEUS clearly demonstrated non-enhancement of the avascular areas within a consolidation, consistent with necrotizing pneumonia or the abscess cavity (Figs. 3 and 4, Online Supplementary Material 1). Also, CEUS showed better demarcation of the extent of a parapneumonic effusion whose appearance on gray-scale US mimicked consolidation (Fig. 5, Online Supplementary Material 2). Distinguishing between these entities is imperative to direct patient care because a complex parapneumonic effusion requires drainage and fibrinolysis.

In all cases in this study, there was high interobserver agreement in interpreting CEUS findings regarding the identification and demarcation of the lung lesions and pleural effusion. In addition, CEUS significantly raised the diagnostic confidence of the observers for diagnosing necrotizing pneumonia. Finally, in addition to improving the initial diagnosis, IV CEUS also proved very useful for following up known complications of pneumonia, without any additional risks [32].

### The role of intracavitary contrast-enhanced ultrasound in pediatric pneumonia

As mentioned, fibrinous strands can form within the infected pleural collection. Initially, these strands are thin, incomplete, and suspend within the pleural fluid. But eventually they organize into thickened septa that increase in number and complexity, creating multiple non-communicating internal loculations. The fluid becomes trapped within these loculations and no longer changes with patient position or respiration to facilitate drainage [41]. In this case, the draining tube might empty the fluid from a loculation while the fibrinous strands completely block its tip or prevent complete drainage of the entire pleural collection. Clinically, the volume of a pleural effusion increases despite the presence of a catheter. Instilling contrast microbubbles into the pleural cavity via the drainage tube helps to identify loculations within the pleural collection and assess the patency of the drain [12]. If there is little to no communication between the multiloculated collections, intrapleural fibrinolysis is indicated to break down the cross-links of these fibrinous strands and resume drainage. Furthermore, follow-up intracavitary CEUS examination can show microbubbles freely moving within the effusion, confirming successful disruption of the fibrin.

## Monitoring response to treatment of pleural-based malignant lesions in children

One pilot study assessed the role of quantitative CEUS performance in 13 children and adolescents with recurrent solid tumors [42]. Primary diagnoses in this study included rhabdomyosarcoma, rhabdoid tumor, Wilms tumor, renal cell carcinoma, hepatocellular carcinoma, osteosarcoma, synovial sarcoma, epithelioid sarcoma and Ewing sarcoma. The investigators used CEUS to monitor the response of these lesions over the course of the anti-angiogenic therapy [42]. CEUS evaluated target lesions prior to, during and at the end of the first course of the children’s therapy. The lesions’ enhancement was quantitatively analyzed using time-intensity curves and the degree of treatment response was assessed.

**Fig. 6:**
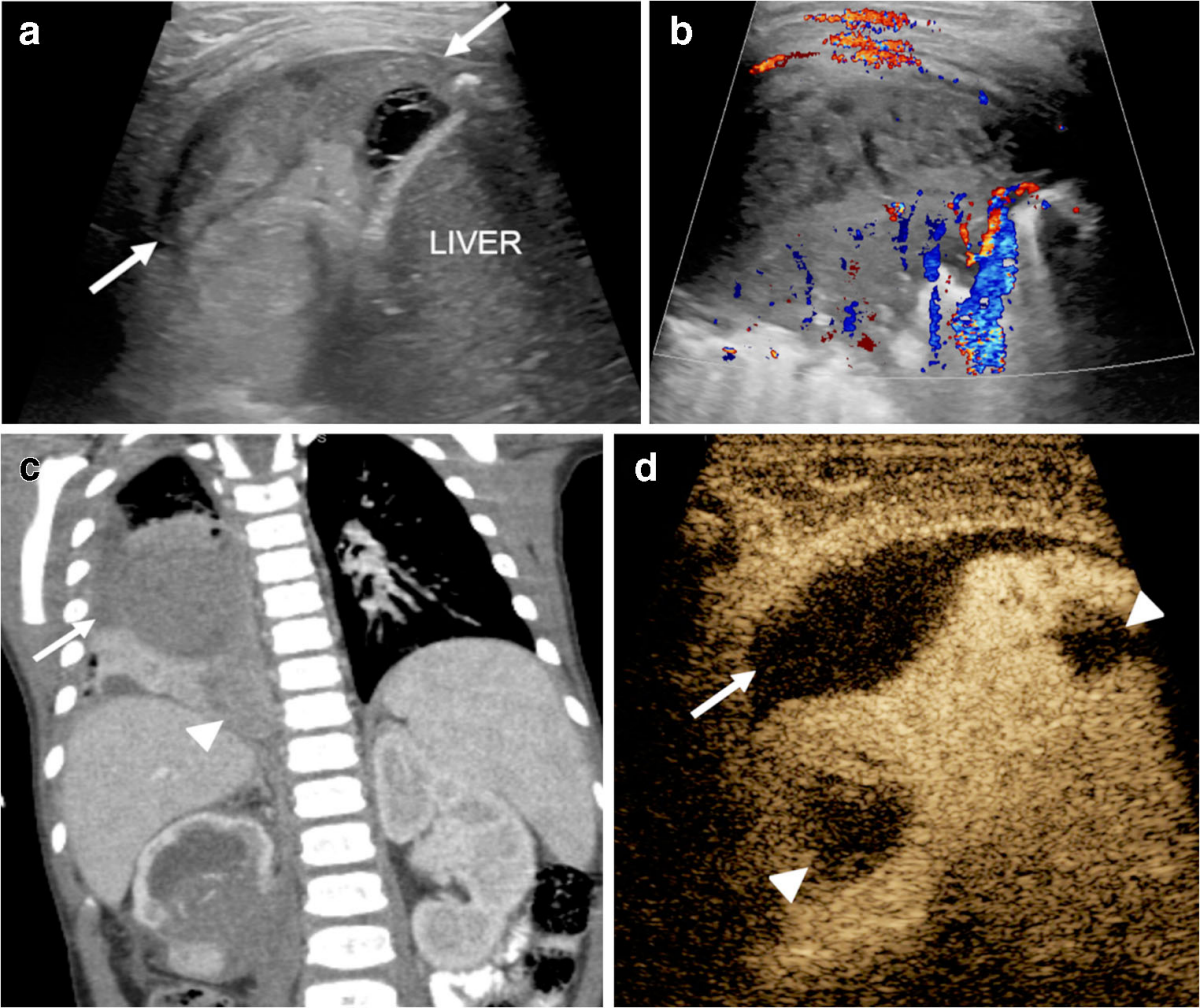
Imaging in a 4.5-year-old girl with history of rhabdoid tumor in the right kidney and lung and pleural metastases. **a** Coronal gray-scale US, transverse view through the right lung base posteriorly, shows a heterogeneous appearance of the right lung base (*arrows*). **b** Coronal color Doppler US shows some blood flow signals in the posterior aspect of the right lung base. **c** Contrast-enhanced chest CT, coronal reformat. The primary mass in the right kidney is demonstrated at the level of the renal pelvis extending into the proximal right ureter. Loculated low-density pleural fluid (*arrow*) is noted. There is enhancing atelectasis in the right lower lobe with a more discrete round heterogeneously hypoattenuating focus inferomedially (*arrowhead*), corresponding to lung metastasis. **d** Contrast-enhanced US, coronal plane. Corresponding to the CT findings, there is heterogeneous enhancement of the right lung with multifocal areas of non-enhancement or hypoenhancement (*arrowheads*) corresponding to necrosis or hemorrhage of the tumor deposits. Other areas of non-enhancement are suspicious for hemorrhagic pleural fluid from tumoral hemorrhage (*arrow*)

Among the quantitative imaging parameters that guided evaluation, peak enhancement, rate of enhancement and area under the curve were used as surrogate imaging markers to depict changes in blood flow. In this study a greater decrease in these imaging parameters was associated with longer time until tumor recurrence. There is considerable scope for confirming these findings in other pleural-based primary malignancies, including infantile fibrosarcoma, pleuropulmonary blastoma, inflammatory myofibroblastic tumors, and subpleural pulmonary metastases from Wilms tumors, neuroblastoma, hepatoblastoma, thyroid carcinoma and osteosarcoma (Fig. 6, Online Supplementary Material 3).

Table 1 summarizes the main established and potential indications for CEUS of the lungs in children.

**Table 1:** Summary of the main established and potential indications for contrast-enhanced ultrasound of the lungs in children

Intravenous	Intracavitary
Established applications • Diagnosing necrotizing pneumonia • Differentiating necrotizing pneumonia from adjacent pleural empyema Potential applications • Directing US-guided biopsies for sub-pleural nodules • Detecting complications post-intervention • Monitoring treatment response of malignant pleural-based lesions	Established applications • Detecting catheter position and confirmation of patency • Identifying fibrous septations forming loculations within a pleural effusion that prevent complete drainage (when instillation of fibrinolytic therapy might be beneficial) • Ensuring resolution of the septations post fibrinolytic administration

## Contrast-enhanced ultrasound for lung applications in adults

The feasibility and added diagnostic value of CEUS for various lung pathologies has been explored in adults [17]. CEUS proved to be capable of discriminating between pulmonary and bronchial arterial vascularity, thus adding more information about the main arterial supply of a lesion than wouldn’t have been available if using only gray-scale lung US or contrast-enhanced CT [14, 15, 17]. The CEUS imaging parameters that might assist in lesion characterization are (1) the time between contrast wash-in and washout, (2) the overall duration of contrast enhancement and (3) the homogeneous or inhomogeneous enhancement pattern of the lesion.

### Pleuropneumonia

A prospective study of 25 people with pleuritic symptoms and focal pleural-based lesions evaluated the use of CEUS to characterize the lesions [14]. While the ages of the patients included in the study were not specified, an imaging example of pleuropneumonia in a 15-year-old boy was provided. All lung lesions in this study were also assessed by contrast-enhanced CT, scintigraphy, radiography or clinical follow-up. Invariably, pleuropneumonia showed quick pulmonary arterial supply that occurred in <6 s as well as pronounced sustained enhancement during the parenchymal enhancement phase (1–5 min). Twelve people in this study had pathological diagnoses other than pleuropneumonia, including pulmonary embolism/infarction, lymphoma, metastasis, granuloma and one unknown lesion. Contrary to the enhancement pattern of pleuropneumonia, these lesions showed a variable pattern with either complete absence of enhancement or delayed and reduced enhancement [14].

### Pulmonary embolism

Another study investigated CEUS for pulmonary embolism by examining adults with confirmed pulmonary embolism and peripheral lung lesions. In most of these cases, CEUS showed absence or inhomogeneous enhancement during the pulmonary arterial phase, suggesting the absence of pulmonary arterial blood supply, without tissue enhancement in the later parenchymal phases [17, 20]. However, in cases of chronic pulmonary or septic embolism, a mixed enhancement was observed, rendering the results of the study inconsistent.

### Atelectasis

A small pilot study investigated how well CEUS could differentiate between compressive and obstructive atelectasis [15]. Initially, the presence of atelectasis was confirmed by CT or radiography. Of the 30 people assessed retrospectively, the 13 with compressive atelectasis showed predominantly pulmonary arterial enhancement and sustained parenchymal enhancement, which persisted after the contrast agent had washed out from the blood pool as compared to splenic enhancement. The CEUS perfusion patterns for obstructive atelectasis were less consistent, with some overlap with the compressive atelectasis group [15, 17]. However, in people in whom central lung cancer was associated with peripheral 'obstructing atelectasis, CEUS was useful for distinguishing between these two entities based on the washout of the central tumor and the sustained enhancement of the atelectasis in the late parenchymal phase. Additionally, CEUS helped to make a distinction between enhancing and non-enhancing tumor tissue and therefore facilitate biopsy.

### Neoplastic vs. non-neoplastic lung pathology

Distinguishing neoplastic from non-neoplastic lung pathology using CEUS proved to be more challenging, with inconsistent enhancement patterns reported in various studies [14–17, 26]. In the most recent and largest adult series so far, 728 people with lung opacities underwent CEUS. Authors concluded that CEUS cannot identify discrete features that allow for a definite distinction between pneumonia and malignant pulmonary lesions [26]. Specifically, there was no difference regarding the onset and duration of enhancement between these types of lesions, and the evaluation of homogeneous or nonhomogeneous distribution of contrast agent within these lesions did not add value.

Similar to these results, a previous study including 137 people compared the enhancement characteristics of malignant (central lung cancer and peripheral malignant lesions) and benign (pneumonia, pulmonary embolus, compressive atelectasis and benign lesions) pleural-based lesions [16]. Although some characteristic enhancement patterns on CEUS were noted among the individual lesions, overall there were no significant differences between benign and malignant lesions. Namely, pulmonary arterial enhancement and sustained parenchymal enhancement were exhibited by all cases of compression atelectasis (caused by either malignant or benign pleural effusion) as well as by the majority (62%) of people with pneumonia. In comparison, absence of pulmonary arterial enhancement and reduced parenchymal enhancement was evident in everyone with pulmonary embolism as well as in the majority (62%) of people with malignant peripheral pleural-based lesions. Benign lung nodules and central lung cancer had no specific patterns of enhancement [16].

Contrary to these findings, other studies reported favorable results using specific CEUS criteria to distinguish infectious and embolic lesions from neoplastic lesions [18, 19]. Heterogeneous enhancement in the first 15 s followed by early washout suggested a neoplastic lesion, whereas infections demonstrated homogeneous enhancement in the first 10 s with late-phase washout [18]. Areas that lacked enhancement represented necrosis or abscess, with reverberation artifacts corresponding to internal gas loculi. The authors reported that the most useful criterion for this differentiation was the timing of washout. Washout time <120 s was indicative of malignant lesions, compared to washout time >120 s, which was most commonly seen in benign lesions. In this study, CEUS provided the correct diagnosis in 95% of examined cases. When the sensitivity for diagnosing neoplasia was compared among CEUS, CT, US and radiography, this study reported that the sensitivity was 95% for CEUS, 97% for CT, 83% for gray-scale US and 87% for conventional radiology [18].

Another series including 95 people with pleural or peripheral lung lesions who underwent CEUS showed that it was 94% sensitive and 95% specific for neoplasia if the enhancement of the lesion started at least 2 s after the enhancement of the normal lung, or simultaneously to the enhancement of the chest wall, liver or spleen [19].

While primary lung malignancy is not common in children, these observations might be useful to assess whether atelectasis is associated with infection, distinguish infection from infarction in critically ill children or in those with sickle cell disease, and potentially differentiate lung lesions such as congenital pulmonary airway malformations and sequestrations from pleuropulmonary blastoma.

## Guiding interventional procedures in pleural-based lesions in adults

Contrast-enhanced ultrasound has been accepted as a complementary technique for US-guided biopsy of subpleural nodules in adults [30]. This technique provided adequate tissue sampling for a conclusive diagnosis in 94.1% of procedures. Even though this study included adults only, the concept of using CEUS-guided biopsy for lung lesions is also applicable in children. Because Wilms tumor, neuroblastoma, hepatoblastoma, papillary thyroid carcinoma and osteosarcoma metastases can all present with subpleural nodules in children, this technique might improve the diagnostic yield during biopsy.

Monitoring for procedure-related complications is another potential use of CEUS. In an adult case report, CEUS was used to show active pleural bleeding during tumor radiofrequency ablation. Real-time CEUS demonstrated contrast extravasation, which was also confirmed angiographically [25]. Identifying intratumoral active hemorrhage following a procedure might also be possible in pediatric patients.

## Conclusion

Although there is limited literature on pediatric pulmonary CEUS applications, the feasibility of this technique for the evaluation of complicated pneumonia and accurate differentiation of necrotizing pneumonia from complex parapneumonic effusion has been demonstrated in children. In addition to these benefits, direct administration of UCA into the pleural cavity can be used to confirm the position of drainage tubes and help decision-making regarding intrapleural fibrinolytic therapy. CEUS can also be used for monitoring response of pleural-based tumors and for procedural guidance and post-procedural monitoring, but these applications must be validated in pediatric patients. The ability of CEUS to distinguish between benign and malignant pleural-based and subpleural lesions remains unclear in adults and has not been studied in children.

## Supplementary Information

Online Supplementary Material 1 Same child as in Fig. 4. Contrast-enhanced US cinematic clip in coronal plane demonstrates a large non-enhancing area in the left lower lobe and a smaller non-enhancing area anteriorly. These findings are in keeping with necrotizing pneumonia (MOV 8087 kb)

Online Supplementary Material 2 Same child as in Fig. 5. Contrast-enhanced US cinematic clip focusing on the left lung base in coronal plane demonstrates homogeneous enhancement of the left lower lobe, with no enhancement of the adjacent complex pleural fluid. Note, there is overall delayed time-to-peak enhancement secondary to cardiac failure (MOV 11680 kb)

Online Supplementary Material 3 Same child as in Fig. 6. Contrast-enhanced US cinematic clip in coronal plane shows heterogeneous enhancement of the right lung, with multifocal areas of hypoenhancement and nonenhancement corresponding to necrosis/hemorrhage of the multiple pulmonary- and pleural-based metastasis and pleural fluid, likely hemorrhagic (MOV 8898 kb)
